# Exclusive Breast-Feeding Practice and Associated Factors among HIV-Positive Mothers in Governmental Health Facilities, Southern Ethiopia

**DOI:** 10.1155/2020/7962054

**Published:** 2020-09-16

**Authors:** Lewam Mebratu, Selamawit Mengesha, Yadessa Tegene, Abraham Alano, Alemayehu Toma

**Affiliations:** ^1^School of Public Health, College of Medicine and Health Science, Hawassa University, Hawassa, Ethiopia; ^2^Policy Study Institute, Hawassa, SNNPR, Ethiopia; ^3^School of Pharmacy, College of Medicine and Health Science, Hawassa University, Hawassa, Ethiopia

## Abstract

**Introduction:**

Globally, over 90% of HIV infections among children are due to mother-to-child transmission and breastfeeding accounts for 5–20% of the burden. Avoidance of inappropriate feeding practices and practicing exclusive breastfeeding is recommended to reduce mother-to-child HIV transmission, but it is hardly practiced. The aim of this study was to determine the prevalence of exclusive breastfeeding practice and associated factors among HIV-positive mothers attending governmental PMTCT clinics in Southern Ethiopia.

**Methods:**

An institution-based cross-sectional study was conducted from April to May 2019. The participants of the study were 209 HIV-positive mothers at the selected PMTCT sites. The study subjects were drawn from 10 health institutions located at 6 towns in Southern Ethiopia which constituted six hospitals and four health centers. Quantitative data were collected using the pretested structured questionnaire. Logistic regression analysis was used to determine the association between the predictors and outcome variable.

**Results:**

Among the 209 participants, 81.6% (95% CI: 75.8–86.5) practiced exclusive breastfeeding and 18.4% (95% CI: 13.5–23.7) practiced mixed feeding. Mothers who had attended the recommended four antenatal visits [AOR: 3.01, 95% CI (1.1–8.28)], who had disclosed their serostatus [AOR: 3.17, 95% CI (1.12–8.99)], who had sufficient knowledge about infant feeding practice [AOR: 3.32, 95% CI (1.15–9.55)], and favorable attitude towards infant feeding practice [AOR: 5.39, 95% CI (1.65–17.6)] were more likely to practice exclusive breastfeeding.

**Conclusion:**

Exclusive breastfeeding was predominantly practiced. But mixed feeding was also being practice considerably. Improving maternal knowledge and attitude towards appropriate infant feeding practice through appropriate counseling on ANC visits could significantly improve EBF practice. It was also evident that promoting disclose of serostatus could empower the mothers to make an informed decision on how to appropriately feed their newborn.

## 1. Introduction

Breastfeeding is one of the most effective ways to ensure child health and survival. If exclusive breastfeeding was practiced at 50 percentage point all over the world, lives of 823 000 (13.8% of deaths) children under 2 years of age would have been saved every year [1]. But, only 40% of infants under six months of age are exclusively breastfed globally [2]. More than half (58%) of children under 6 month of age were exclusively breastfed in Ethiopia in 2016, but the percentage of exclusive breastfeeding declines with age from 74% at 0-1 months to only 36% at 4-5 months of age [3].

Breastfeeding becomes a source of worry when it carries the risk of exposing the child to harmful drugs or infections, such as human immune deficiency virus (HIV). With no intervention, about a third of HIV-positive women will transmit the virus to their children during pregnancy, delivery, and breastfeeding, and breastfeeding accounts for 5 to 20% of the transmission [4]. Globally, over 90% of the HIV infections among children are due to mother-to-child transmission (MTCT) and nearly 90% of the HIV-infected children are living in sub-Saharan Africa [5]. By 2018, there were about 690 000 people living with HIV in Ethiopia [6]. Based on a point estimate in 2007, 75420 pregnant women and 64000 children under the age of 14 years were estimated to be HIV-positive in the country. And, sadly over 10000 HIV-infected children died in 2007 alone [7].

In order to prevent MTCT of HIV during breastfeeding while ensuring provision of appropriate nutrition to the child, the World Health Organization (WHO) developed a guideline on infant and young child feeding (IYCF) which was improved over the years. The 2010 guideline states that “in settings where health services provide and support lifelong ART, including adherence counseling, and promote and support breastfeeding among women living with HIV, the duration of breastfeeding should not be restricted. Mothers known to be HIV-infected should exclusively breastfeed their infants for the first 6 months of life, introducing appropriate complementary foods thereafter and continue breastfeeding. And breastfeeding should then only stop once a nutritionally adequate and safe diet without breast milk can be provided.” This recommendation aims to reduce MTCT of HIV as well as mortality due to other causes such as infections and malnutrition. Thus, the focus is now on ensuring HIV-free survival and not just on preventing the transmission of HIV [8, 9].

Although this is the general recommendation, the level of EBF practice has been shown to be generally low. According to some studies conducted in Ethiopia, a proportion of exclusively breastfeed HIV-exposed infants (HEIs) widely varies among regions with the highest value of EBF being 90% (in Tigray) and the lowest being 30.6% (in Addis Ababa) with ERF of 3.4% and 47% for the regions, respectively [10, 11]. The prevalence of EBF among HIV-positive mothers in SNNPR has been shown to be around 50% [12, 13].

Alongside the low level of EBF, inappropriate feeding practices such as mixed feeding are practiced significantly. It is practiced as high as 35.6% among HIV-positive mothers in some regions of Ethiopia [12]. Mixed feeding is said to result in a significantly higher risk of acquiring infection than exclusive breastfeeding [14, 15], and it has been shown to be triggered by several factors, such as cultural pattern or influence of the social norm and general knowledge or specific knowledge about benefits and risks of infant feeding practice (IFP) in the case of HIV, which is strongly related to counseling given during antenatal care (ANC) and postnatal care (PNC) visits and disclosure of HIV status to name but few [10–13].

Antenatal care (ANC) service, provided at health facilities, is one of the key contact areas to provide numerous health information, counseling, and guidance to create better knowledge and attitude towards IFP [12, 16] but it was reported that only 32% of women in Ethiopia had at least four ANC visits during their last pregnancy, while 37% had none, limiting the impact that can be made during ANC follow-ups [3].

Therefore, this study aims to assess the prevalence of EBF and factors associated with it among HIV-positive mothers attending PMTCT clinics in Southern Ethiopia.

## 2. Methods

### 2.1. Study Design and Period

A facility-based cross-sectional study with quantitative approaches through an interviewer-administered questionnaire was conducted from April 8 to May 10, 2019.

### 2.2. Study Setting and Population

There are 13 specific localities in the region that are identified to be disproportionately affected by the HIV epidemic [17]. Out of these, 6 localities which have 10 health institutions with a relatively larger number of eligible participants were included in the study.

The source population of the study was all HIV-positive mothers with a child age below 18 months attending governmental PMTCT clinics in southern Ethiopia.

The study population comprised HIV-positive mothers with the child age below 18 months, who attended the PMTCT clinics in the selected governmental health institutions during the study period.

### 2.3. Inclusion and Exclusion Criteria

HIV-positive mothers with the child age below 18 months, attending PMTCT clinics at the selected governmental health institutions, were included. Those mothers who were unable to respond due to communication barriers other than language barrier were excluded.

### 2.4. Sample Size Determination

The sample size was calculated using the formula for a single population proportion by using the hypothesized proportion of EBF 56.3% [12], 5% margin of error, and 95% level of confidence. Accordingly, the sample size required for the first specific objective was 378 participants.

From the 10 PMTCT sites (6 hospitals and 4 health centers), a sampling frame of 325 mothers with a child below the age of 18 months was obtained from registration books of each health facility. Taking this figure, the sample size is corrected to 175. Adding 15% for nonresponse, the grand total sample size required was 201 HIV-positive mothers with children under 18 months of age who attend PMTCT service in selected health facilities.

Factors such as the educational status of the mother, ANC follow-up, and attitude towards infant feeding options have been shown to be independent factors affecting EBF [10, 12, 13]. Based on these identified factors, sample size was calculated for each factor. Because all the calculated sample sizes are smaller than that calculated ones for the first objective, the larger sample size of 201 was taken as the final sample size.

### 2.5. Operational Definitions

#### 2.5.1. Exclusive Breastfeeding

The infant receives breast milk (including expressed breast milk or breast milk from a wet nurse) and allowed to receive ORS, drops, and syrups (vitamins, minerals, and medicines), but nothing else [18].

#### 2.5.2. Early Cessation of Breastfeeding

Discontinuing breastfeeding before the child reaches 12 months of age [18].

#### 2.5.3. Knowledge about MTCT and PMTCT

Those who mentioned at least two out of four correct transmission methods as well as at least two out of four correct prevention methods were considered as having sufficient knowledge [16].

#### 2.5.4. Knowledge about IFP

The participants who correctly answered 4 out of 7 questions were considered as having sufficient knowledge and values below that were considered as insufficient knowledge [19].

#### 2.5.5. Attitude towards IFP

It was assessed by employing seven questions with 5-point Likert scale. The mean of sum of scores was computed, and values below 27 were considered as having unfavorable attitude and values 27 and above were considered as having favorable attitude.

### 2.6. Sampling Procedure and Technique

Eighteen health institutions were selected based on the provision of PMTCT service. Five health facilities were excluded from the study because they had less than 10 clients at their PMTCT clinics. Additional 3 sites were also excluded for the pretest purpose. One month time was allocated for the data collection period with the aim of attaining a minimum of 201 participants. In order to increase the sample size as much as possible with in the allocated data collection period, all eligible mothers who visited the health institutions were consecutively included. By consecutively enrolling all the eligible and willing clients, 211 participants were included by the end of the allocated study period.

### 2.7. Data Collection Tools and Procedures

A structured questionnaire was prepared by reviewing WHO guidelines and other researches conducted on similar topics were used to collect the required data [13, 18–21]. It was prepared originally in English and then forward translated into Amharic by a health professional familiar with the terminologies used. Back translation was performed by an individual who was fluent both in English and Amharic. The pretest of the tool was conducted on 5% of the final sample size out of the study area.

Three-day intensive training was given to data collectors and supervisors on the objective of the study, the confidentiality of information, and the techniques for conducting the interview. Data collectors were pharmacists working at ART pharmacies of the health facilities as they are already aware of the participant's serostatus but are less likely to introduce bias to the information gathered.

The principal investigator, 10 pharmacists as data collectors, and 4 health officers as supervisors were involved in the study. All the eligible clients who visited the health facility during the study period were told about the purpose of the study and were asked to participate. Those who accepted were asked to verify their willingness by signing the consent form, and the interview began afterwards.

### 2.8. Statistical Analysis

All the questionnaires were checked for error and coded. Out of 211 questionnaires, 2 were incomplete and they were discarded. Data of 209 participants were entered into Epi info version 7 software programs. SPSS version 20 software program was used for cleaning and analyzing the data. Mean (SD), frequency, and percentages were used to describe different characteristics of the participants and the prevalence of the outcome variable, EBF.

The bivariable analysis was conducted to examine the association between the dependent and each independent variable. During cross-tabulation, marital status and having at least one ANC follow-up had cells with values less than 5 and only had two categories; therefore, they were excluded from multivariable analysis. All variables with a *p* value <0.25 [22] during the bivariable analysis were further fitted to multivariable analysis and *p* value less than 0.05 was taken as statistically significant.

## 3. Results

### 3.1. Sociodemographic Characteristics of the Participants

A total of 209 HIV-positive mothers with a child below 18 months of age were included in our study. The mean age (SD) of the mothers was 29 (4.97) years, ranging from 18 to 45 years. Majority of the study participants (73.2%) were urban residents while 82% of the mothers were married or living with a spouse. The study also showed that larger proportion of the mothers (48.3%) had only primary education and moreover similar to the mothers, primary education was the highest educational level attained by their husbands. As indicated in Table 1, 64.6% of the study participants were housewives, and unfortunately, it was only nearly half of the participants (49%) who earns a monthly income of above 1000 ETB (roughly 29$). In this study, half (51%) of the mothers were found to have two or more children, of which 31% of them had their last child being aged below 6 months and 57.4% of the offspring were of male sex (Table 1).

### 3.2. Health Care Utilization and Health Condition

Ninety-three percent of our study participants have attended ANC follow-up, of which majority of them (69%) had attended more than four ANC visits and 92% of them delivered at a health facility. Eighty percent of the mothers reported that they have received counseling about infant feeding methods during their visits for ANC, PNC, delivery, and/or ART services. After their delivery, 86% of the mothers revisited the health facilities for a PNC follow-up, and only a few reported to have experienced breast related and/or obstetric problems but a quarter of them reported child illness since birth (Table S1).

### 3.3. Knowing and Disclosure of Serostatus

More than 70% of the mothers knew their serostatus before their last pregnancy. Out of these, 55% of them disclosed their status to their husband and 46% disclosed to their family. When asked about their husbands, 81% of the mothers reported that their husbands also knew their serostatus.

### 3.4. Mother's Knowledge on MTCT and PMTCT of HIV/AIDS

The majority (80%) of the mothers correctly identified at least two MTCT routes and two methods of reducing transmission. Similarly, 80% of the mothers correctly responded to four out of seven of the knowledge-based questions about IFP and 82% had a favorable attitude towards IFP (Table 2).

### 3.5. Infant Feeding Practice

Out of the total 209 mothers, nearly all except two (207) had ever breastfed their infants and the two mothers who opted for ERF both mentioned fear of MTCT of HIV as their sole reason for not choosing to breastfeed. Of the mothers who chose to breastfeed their children, 81.6% of them with 95% CI (75.8–86.5) practiced EBF while the rest 18.4% with 95% CI (13.5–23.7) practiced mixed feeding. During the study period, twenty-seven percent of the mothers had already stopped breastfeeding, among which 32% of the mothers stopped breastfeeding before their children turned the age of one year. The repeatedly mentioned reasons for early cessation of breastfeeding practice were fear of MTCT of HIV and advice from health professionals.

As shown in Table 3, majority of the mothers provided colostrum for their infants and did not provide anything before the initiation of breastfeeding, but 29% of them initiated breastfeeding after one hour of delivery. One-hundred forty-three mothers have already initiated complementary feeding at the time of the study, while 17% of them initiated the practice before the child reached 6 months of age (Table 3).

### 3.6. Factors Associated with Exclusive Breastfeeding Practice

The bivariable analysis showed EBF practice to have significant associations with place of residence, occupation, household income, antenatal and postnatal visits, place of delivery, receiving counseling on how to breastfeed during maternal or child illness, disclosure of HIV status, knowledge about MTCT, PMTCT and IFP, and attitude towards IFP. In multivariable analysis, ANC follow-up, disclosure of HIV status, and knowledge about and attitude towards IFP maintained their significant association with EBF practice.

The odds of exclusively breastfeeding their child was 3 times higher for mothers who attended the recommended four ANC visits to the health facilities than their counterparts [AOR: 3.01, 95% CI: (1.01–8.28)]. Those mothers who had disclosed their serostatus to either their husband, friends, family, or the community in general were 3 times more likely to EBF their child than those who kept their serostatus hidden [AOR: 3.17, 95% CI: (1.12–8.99)]. Similarly, mothers who had sufficient knowledge about IFP were 3 times more likely to practice EBF than those who had insufficient knowledge [AOR: 3.32, 95% CI: (1.15–9.55). As can be seen in Table 4, the odds of practicing EBF was 5 times more likely for the mothers who had a favorable attitude towards IFP than their counterparts [AOR: 5.39, 95% CI: (1.65–17.56)] (Table 4).

## 4. Discussion

The study was done to assess the practice of EBF and factors affecting it among HIV-positive mothers with a child under 18 months of age. Out of a total of 209 participants, 81.6% [95% CI (75.8–86.5)] practiced EBF. This prevalence was comparable with that other studies conducted in the different regions of the country: Oromia (85.5%) [19], Gondar (83.7%) [23], and Gojjam (77.1%) [24]. However, the prevalence is higher than that of the similar studies conducted in the same region (SNNPR) at different times of 2011 and 2013 [12, 13] which recorded a prevalence of 56.3% and 48.2%, respectively. The discrepancy might be ascribed to the difference in time of conducting the study during which there has been an improvement in health care utilization and knowledge about MTCT.

The Ethiopian demographic and health survey reported that there has been 28% increment in ANC service utilization and 7% increment in knowledge of women about the ability to reduce MTCT of HIV by medications from 2011 to 2016 [3]. This, in turn, reduces avoidance of breastfeeding due to fear of transmitting the infection to the child which was the reason mentioned in our study by both women who avoided breastfeeding and also by the group of mothers who discontinued to breastfeed before the age of 6 months (44%).

In a study conducted in Mekelle Ethiopia, 90.3% of the participants reported to exclusively breastfeed their infants. This higher value might be due to the higher level of knowledge about MTCT of HIV (83.1% vs. 79.4%) and disclosure of HIV serostatus (90.3% vs. 72.2%) reported by the study. Improved knowledge about MTCT of HIV has been shown to increase the practice of EBF. Similarly, disclosure of HIV serostatus has been shown to positively affect EBF practice [10, 11].

The proportion of mixed feeding in this study was 18.4% [95% CI (13.5–23.7)] which is comparable with that of the study performed in Addis Ababa (15.3%) [11] and Gojjam (14.2%) [24]. Probably for similar reasons mentioned previously, the above studies conducted in SNNPR reported a higher prevalence of 35.6% and 34.6%, respectively [12, 13]. A lower prevalence of 6.3%, 8.3%, and 10.5% were reported in studies performed in Mekelle, Oromia, and Gondor, respectively. Some of these studies mentioned societal influences and perception of inadequacy of breast milk as reason for mixed feeding [10, 23].

The rate of ERF was very low in this study (1%), and this value is similar to the finding of the study conducted in Mekelle (3.4%) [10]. This might be due to low economic status of the mothers who might not afford formula milk and the other possible reason might be breastfeeding is the recommended way of practice by health professionals.

Mothers who received the recommended four ANC/PMTCT visits were 3.7 times more likely to practice EBF. Similar results were reported in studies conducted in Addis Ababa, SNNPR, and Gojjam [12, 16, 24]. Among the four prongs of PMTCT service, the third prong deals with the prevention of HIV transmission from infected women to their infants, and the national strategy to accomplish this task entails activities related to the provision of ART prophylaxis or medication to the mother and child, safe obstetric care, and provision of appropriate counseling on infant feeding and support exclusive breastfeeding [4]. The appropriate practice of the PMTCT guideline ensures that all mothers who attended the service will get counseling about MTCT and PMTCT of HIV and IFP. EDHS reports showed that there has been a marked increase in the uptake of these services from 34% (2011) to 62% (2016) for any ANC service and from 19% (2011) to 32% (2016) for the recommended four visits, but the above figures also shows that nearly forty percent of pregnant women still did not attend any ANC visit [3].

This study also showed that majority of the participants had sufficient knowledge about MTCT, PMTCT, and IFP (80%) as well as favorable attitude towards IFP (82%). The findings demonstrated those mothers who had good knowledge about IFP were 3.6 times more likely to practice EBF. Similarly those who had favorable attitude towards IFP were 5 times more likely to practice EBF than their counterparts. These results are additional evidences that show improving the knowledge and attitude of the mothers significantly impacts their practice. Some studies suggest lack of full information regarding advantage and disadvantage of feeding options and access of mother support group as a source of unfavorable attitude towards EBF [10, 12].

A study conducted in Addis Ababa showed when the husbands are not aware of the HIV status of their wives, they will likely impose inappropriate infant feeding practice and if they refuse they will suspect their wives to have HIV and which in turn results in dispute [11]. This fact is also supported by the results of this study where the women who disclosed their status were 3 times more likely to practice EBF than those who did not disclose their status. Other studies conducted in different parts of the region consistently showed that disclosure of HIV status was significantly associated with EBF practice [10, 12, 23, 24]. The reasons for not disclosing their status were described, in a study conducted in Addis Ababa, to be the low economic status and economical dependency of the mothers on their husbands. They fear of outcomes like divorce, physical violence, and having to raise a child alone. Disclosing their status also has the benefit of not having to hide while formula feeding and it enables them to have favorable attitude towards EBF [11, 13].

### 4.1. Limitation of the Study

The study was health facility based, and the findings may not be generalizable to all HIV-positive mothers with HEIs. A relatively smaller sample size was used. This was because very small numbers of HIV-positive mothers with HEIs attend health facilities, and it is difficult to trace them at the community level. In order to improve the sample size, 10 health institutions from six cities were included rather than the previously intended 5 health institutions from one city. Although some responses might be affected by recall errors, effort was taken to minimize it by employing multiple questions to assess one outcome.

## 5. Conclusion

This study revealed that majority of the mothers practiced EBF. Considering the risk of HIV transmission, fairly large number of mothers practiced mixed feeding. Major determinants of EBF practice were found to be attending the recommended four ANC visits, sufficient knowledge about IFP, having favorable attitude towards IFP, and disclosing serostatus. ANC and PMTCT services have been shown to be an entry point to provide cocktail of services. Although improvements were noted on utilization of these services, the figure is far from satisfactory. Improving utilization could positively affect the mothers' knowledge and attitude towards appropriate IFP which in turn affects EBF practice. Counseling about IFP was shown to be mainly focused on EBF, and all other options with their advantages and disadvantages should be clearly communicated with the mothers. Creating favorable environment for the mothers to openly disclose their serostatus could enable them to choose appropriate IFP and practice it with freedom.

## Figures and Tables

**Table 1:** Sociodemographic characteristics of HIV-positive mothers with a child below 18 months, SNNPR, Ethiopia, 2019.

Variable (*n* = 209)	Categories	Frequency	Percent
Age of the mother (years)	18–24 yrs	26	12.4
	25–29 yrs	66	31.6
	30–34 yrs	67	32.1
	35 + yrs	50	23.9

Current place of residence	Rural	56	26.8
	Urban	153	73.2

Religion	Christian	186	89
	Muslim	21	10
	Others	2	1

Marital status	Never married	14	6.7
	Married and living with a spouse	171	81.8
	Others	24	11.5

Maternal education	No formal education	37	17.7
	Primary (1–8 grades)	101	48.3
	Secondary (9–12 grades)	46	22
	Tertiary and above	25	12

Husband education (*n* = 171)	No formal education	27	15.8
	Primary (1–8 grades)	64	37.4
	Secondary (9–12 grades)	44	25.7
	Tertiary and above	36	21.1

Occupation	Housewife	135	64.6
	Work outside the house	74	35.4

Household income	≤500	64	30.6
	501–1000	43	20.6
	≥1001	102	48.8

Number of living children	1 child	48	23
	2 children	54	25.8
	More than 2 children	107	51.2

Age of the last child	Less than 6 month	65	31.1
	6–11 month	58	27.8
	12–17 month	86	41.1

Sex of the last child	Male	120	57.4
	Female	89	42.6

Marital status: others (separated, divorced, or widowed); tertiary and above: diploma, degree, and vocational.

**Table 2:** Mother's knowledge on MTCT and PMTCT of HIV-positive mothers with a child below 18 months of age, SNNPR, Ethiopia, 2019.

Variable (*n* = 209)	Frequency	Percent
Knowledge of MTCT and PMTCT		
Poor knowledge	43	20.6
Good knowledge	166	79.4

Knowledge about infant feeding practice		
Poor knowledge	42	20.1
Good knowledge	167	79.9

Attitude towards infant feeding practice		
Unfavorable attitude	37	17.7
Favorable attitude	172	82.3

MTCT: mother-to-child transmission, PMTCT: prevention of mother-to-child transmission.

**Table 3:** Infant feeding practice of HIV-positive mothers with a child below 18 months of age, SNNPR, Ethiopia, 2019.

Variables	Categories	Frequency	Percentage (95% CI)
Ever breastfed^*∗*^	Yes	207	99
	No	2	1
Still breastfeeding^*∗∗*^	Yes	151	72.9
	No	56	27.1
Age at breastfeeding cessation^*∗∗*^	Early cessation	18	32.1
	Timely cessation	38	67.9
Reason for early cessation of breastfeeding^*∗∗*^ (more than one answer is possible)	To encourage the child to take other food items	4	22.2
	Fear of MTCT of HIV	8	44.4
	Advised by a health professional	8	44.4
Initiation of breastfeeding^*∗∗*^	Timely initiated	147	71
	Late initiation	60	29
Colostrum feeding^*∗∗*^	Yes	192	92.8
	No	15	7.2
Prelacteal feeding^*∗∗*^	Yes	14	6.8
	No	193	93.2
Mixed feeding^*∗∗*^	Yes	38	18.4 (13.5–23.7)
	No	169	81.6
Complimentary feeding^*∗*^	Yes	143	68.4
	No	66	31.6
Age at complementary feeding	5 month or below	25	17.5
	6th month	97	67.8
	7 months or above	21	14.7
Lifelong exclusive breastfeeding^*∗∗*^	Yes	169	81.6 (75.8–86.5)
	No	38	18.4

^*∗*^Calculated among all 209 participants, ^*∗∗*^calculated among 207 participants who ever breastfed; MTCT: mother-to-child transmission.

**Table 4:** Factors associated with EBF practice of mothers with a child under 18 months of age attending in SNNPR, Ethiopia, 2019.

Variables (*n* = 207)	EBF		COR (95% CI)	AOR (95% CI)
	Yes	No		
Place of residence				
Urban	135	16	5.46 (2.59–11.51)	2.1 (0.72–6.08)
Rural	34	22	1	1

Occupation				
Work outside the house	65	8	2.34 (1.01–5.43)	1.39 (0.43 – 4.49)
House wife	104	30	1	1

Monthly household income				
≤500	45	19	1	1
501–1000	35	7	0.32 (0.14–0.72)	1.8 (0.4–8.09)
≥1001	89	12	0.67 (0.25 –1.85)	1.01 (0.31–3.25)

Recommended ANC visit (*n* = 195)				
Yes	124	11	5.22 (2.3–11.89)	3.01 (1.1–8.28)^*∗*^
No	41	19	1	1

Place of delivery				
Health facility	161	29	6.25 (2.23–17.52)	1.77 (0.29–10.63)
At home	8	9	1	1

Postnatal care follow-up				
Yes	151	27	3.42 (1.45–8.03)	1.07 (0.3–3.91)
No	18	11	1	1

Counseling on breastfeeding during child and maternal illness				
Yes	111	14	3.28 (1.58–6.82)	1.14 (0.39–3.29)
No	58	24	1	1

Knowledge of MTCT and PMTCT				
Sufficient knowledge	145	20	5.44 (2.52–11.74)	2.07 (0.7–6.17)
Insufficient knowledge	24	18	1	1

Disclosure of HIV status				
Yes	135	13	7.64 (3.54–16.47)	3.17 (1.12–8.99)^*∗*^
No	34	25	1	1

Knowledge about IFP				
Sufficient knowledge	147	18	7.42 (3.41–16.17)	3.32 (1.15–9.55)^*∗*^
Insufficient knowledge	22	20	1	1

Attitude towards IFP				
Favorable attitude	150	21	6.39 (2.88–14.19)	5.39 (1.65–17.6)^*∗*^
Unfavorable attitude	19	17	1	1

^*∗*^Statistically significant at *p* value <0.05; 1: the reference category. ANC: antenatal care, MTCT: mother-to-child transmission, PMTCT: prevention of mother-to-child transmission, IFP: infant feeding practice.

## Data Availability

The data that support the findings of this study will be available from the corresponding author upon reasonable request in the form of statistical package for social sciences (SPSS) spread sheet.

## Abbreviations

AIDS: Acquired immune deficiency syndrome
ANC: Antenatal care
ARV: Anti-retroviral
EBF: Exclusive breastfeeding
ERF: Exclusive replacement feeding
HIEs: HIV-exposed infants
HIV: Human immunodeficiency virus
IYCF: Infant and young child feeding
MF: Mixed feeding
MOH: Ministry of Health
MTCT: mother-to-child transmission
PNC: Postnatal care
PMTCT: Prevention of mother-to-child transmission of HIV
WHO: The World Health Organization.

## Supplementary Materials

Supplementary Materials Supplementary Table S1: health care utilization and health condition of HIV-positive mothers with a child below 18 months, SNNPR, Ethiopia, 2019. Supplementary Table S2: knowing and disclosure of serostatus of HIV-positive mothers with a child below 18 months, SNNPR, Ethiopia, 2019.
