# Supplementary material for: Exclusive Breast-Feeding Practice and Associated Factors among HIV-Positive Mothers in Governmental Health Facilities, Southern Ethiopia
Source: J Nutr Metab. 2020 Sep 16;2020:7962054. doi: 10.1155/2020/7962054 (PMC7519186; doi:10.1155/2020/7962054)
Supplement: Supplementary Materials — Supplementary Table S1: health care utilization and health condition of HIV-positive mothers with a child below 18 months, SNNPR, Ethiopia, 2019. Supplementary Table S2: knowing and disclosure of serostatus of HIV-positive mothers with a child below 18 months, SNNPR, Ethiopia, 2019. [file 7962054.f1.doc]

**Table S1:**  Health care utilization and health condition of HIV-positive mothers with a child below 18 months, SNNPR, Ethiopia, 2019

| **Variable (n=209)** | **Categories** | **Frequency** | **Percent** |
| --- | --- | --- | --- |
| Antenatal care follow-up | Yes | 195 | 93.3 |
|  | No | 14 | 6.7 |
| Number of antenatal care visits (n=196) | <4 visits | 60 | 30.6 |
|  | 4 or more visits | 135 | 69.2 |
| Place of delivery | Health facility | 192 | 91.9 |
|  | At home | 17 | 8.1 |
| Mode of delivery | Cesarean section | 27 | 12.9 |
|  | SVD | 182 | 87.1 |
| Counseling about infant feeding methods | Yes | 167 | 79.9 |
|  | No | 42 | 20.1 |
| Time of counseling* | Antenatal care | 140 | 83.8 |
|  | Delivery | 78 | 46.7 |
|  | Postnatal care | 76 | 45.5 |
|  | ART | 79 | 47.3 |
| Counseling was sufficient to make informed decision (n=167) | Yes | 157 | 94 |
|  | No | 10 | 6 |
| Postnatal care follow up | Yes | 180 | 86.1 |
|  | No | 29 | 13.9 |
| Breast related problems | Yes | 23 | 11 |
|  | No | 186 | 89 |
| Obstetric problems | Yes | 13 | 6.2 |
|  | No | 196 | 93.8 |
| Child ill since birth | Yes | 68 | 32.5 |
|  | No | 141 | 67.5 |

SVD- Spontaneous vaginal delivery, * more than one answer is possible

Table S2**:** Knowing and disclosure of serostatus of HIV-positive mothers with a child below 18 months, SNNPR, Ethiopia, 2019

| **Variable (n=209)** | **Frequency** | **Percent** |
| --- | --- | --- |
| Time of knowing the serostatus | |  |
| Before the last pregnancy | 155 | 74.2 |
| During the last pregnancy | 32 | 15.3 |
| During last delivery | 13 | 6.2 |
| After last delivery | 9 | 4.3 |
| Husband tested for HIV (n=195) |  |  |
| Yes | 158 | 81 |
| No | 15 | 7.7 |
| Didn't know | 22 | 11.3 |
| Disclosure of HIV status |  |  |
| Yes | 151 | 72.2 |
| No | 58 | 27.8 |
| Status disclosed to* |  |  |
| Husband/spouse | 114 | 54.5 |
| Family | 96 | 45.9 |
| Friend | 44 | 21.1 |
| Community | 28 | 13.4 |

* More than one answer is possible
